# Activity and expression of ADP-glucose pyrophosphorylase during rhizome formation in lotus (*Nelumbo nucifera* Gaertn.)

**DOI:** 10.1186/s40529-016-0140-z

**Published:** 2016-09-30

**Authors:** Libao Cheng, Xian Liu, Jingjing Yin, Jianqiu Yang, Yan Li, Linchong Hui, Shuyan Li, Liangjun Li

**Affiliations:** 1 School of Horticulture and Plant Protection of Yangzhou University, Yangzhou, Jiangsu China; 2 College of Guangling, Yangzhou University, Yangzhou, Jiangsu China

**Keywords:** Lotus root, ADP-glucose pyrophosphorylase, Starch, *NnAGP*, Expression

## Abstract

**Background:**

Lotus root is a traditional and popular aquatic vegetable in China. Starch is an important component of the rhizome and directly affects the quality of processed products. ADP -glucose pyrophosphorylase (AGPase) is a rate-limiting enzyme associated with starch biosynthesis in plants. Therefore, in the present study, AGPase activity and *NnAGP* expression during rhizome development of lotus were analyzed.

**Results:**

Among 15 cultivars analyzed, the contents of amylose and total starch in the rhizome were highest in ‘Mei Ren Hong’. ‘Su Zhou’ and ‘Zhen Zhu’ showed the lowest amylose, amylopectin and total starch contents. In the rhizome, activity of AGPase was highest at the middle swelling stage of development, and higher activity was observed in the ‘Hou ba’ leaf and terminational leaf at the same stage. Three AGPase genes, comprising two large subunit genes (*NnAGPL1* and *NnAGPL2*) and one small subunit gene (*NnAGPS*), were isolated and identified. The deduced amino acid sequences showed 40.5 % similarity among the three genes. Full-length genomic DNA sequences of *NnAGPL1*, *NnAGPL2*, and *NnAGPS* were 4841, 11,346 and 4169 bp, respectively. Analysis of the temporal and spatial expression patterns revealed that the transcription levels of *NnAGPL1* and *NnAGPS* were higher in the rhizome, followed by the ‘Hou ba’ leaf, whereas *NnAGPL2* was significantly detected in the ‘Hou ba’ leaf and terminational leaf. The initial swelling stage of rhizome development was accompanied by the highest accumulation of mRNAs of *NnAGPL1*, whereas expression of *NnAGPL2* was not detected during rhizome development. The transcript level of *NnAGPS* was highest at the initial swelling stage compared with the other rhizome developmental stages. Transcription of *NnAGPL1*, *NnAGPL2*, and *NnAGPS* was induced within 24 h after treatment with exogenous sucrose. The mRNA level of *NnAGPL1* and *NnAGPS* was increased by exogenous ABA, whereas transcription of *NnAGPL2* was not affected by ABA.

**Conclusions:**

The three AGPase genes display marked differences in spatial and temporal expression patterns. Regulation of AGPase in relation to starch synthesis in lotus is indicated to be complex.

**Electronic supplementary material:**

The online version of this article (doi:10.1186/s40529-016-0140-z) contains supplementary material, which is available to authorized users.

## Background

Lotus (*Nelumbo nucifera* Gaertn.) belongs to the dicot family Nelumbonaceae, which shows many features of monocot plants, and is a popular aquatic herb vegetable (Xue et al. 2012). Lotus originates from India and China, and is widely cultivated in China, Japan, and other Southeast Asian countries for multiple purposes (Sakamoto 1977). The processed products of the rhizome (often termed lotus root) are in great demand in many countries, including Japan, Korea, the United States and European countries, as a type of off-season vegetable. Lotus root products, including drinks, teas, lotus seeds, fresh, salted, and boiled rhizomes, and lotus root starch, are extremely popular in the daily diet because these products are rich in starch, proteins, vitamins, and minerals (Liu et al. 2010; Slocum and Robinson 1996). In addition, nodus Nelumbinis Rhizomatis, germ, stamens, and lotus root stems are important ingredients in traditional medicine (Borgi et al. 2007; Renato et al. 2007; Terashima et al. 2011).

Starch is the most important component present in the rhizome and comprises an average content of 10–20 % of the total fresh weight of the rhizome, but the percentage varies among cultivars. The content and form of starch are critical factors in determining the value and storage of starch-based food and industrial products (Mu-Forster et al. 1996). The structure of the starch granule is complex with two distinct types of polysaccharidic components. The first type is amylose, which consists of many unbranched α-(1,4)-linked glucan chains. The enzyme ADP-glucose pyrophosphorylase (AGPase) mediates the biosynthesis of α(1 → 4) glucans (Preiss 1988). It catalyzes the synthesis of ADP-glucose from glucose-l-phosphate and ATP in leaves and storage organs (Miiller-Rober et al. 1990). The second type of polysaccharide is amylopectin, which is formed by a large number of straight glucan chains with branching points at α(1 → 6) linkages (Le et al. 2010; Vlachos and Karapantsios 2000). The ratio of amylose to amylopectin is the main factor that determines the properties of starch in cultivated species (Fredriksson et al. 1998). In some plants, the starch content in the developing rhizome can be improved by means of variation in the amylose:amylopectin ratio (Policegoudra and Aradhya 2008; Zobel 2006).

The many cultivars of lotus can be classified into two broad categories on the basis of the starch content of the rhizome. The main features of the first category include crispiness owing to low starch content, high water and sugar content, and high crude fiber content. These characteristics often result in precipitation of starch, gelatinization, and low viscosity during product processing, which are responsible for the crispiness, refreshing taste, and good sensory quality of the rhizome. Products of this type, such as salted, frozen, or boiled lotus root, are exported worldwide because of the long shelf life and convenient transportation. The second category of lotus cultivars is characterized by high starch content and low water content. The high content of granular starch gives the rhizome a soft texture, pliability, and increases the viscosity of root-derived products (Wattebled et al. 2002).

In most plants, ADP-glucose pyrophosphorylase is considered to be a key regulatory enzyme in starch synthesis (Laughlin et al. 1998). The enzyme regulates the first step of starch synthesis (Slattery et al. 2000). Starch content is significantly decreased in the endosperm in the *shrunken*-*2* (*sh2*) mutant of maize, which shows low activity of AGPase, thus indicating the importance of AGPase in starch synthesis (Bhave et al. 1990; Singletary et al. 1997). The *Sh2* locus encodes the large subunit of endosperm AGPase (Bhave et al. 1990). The subunit composition of AGPase differs among plant species. The two subunits of AGPase isolated from spinach leaves migrate as 51 and 54 kD proteins, and show major structural differences (Morell et al. 1987). In potato tubers, AGPase is composed of two subunits of similar molecular weight (Sowokinos and Preiss 1982). In maize endosperm, AGPase consists of four subunits (Plaxton and Preiss 1987).

Four types of enzymes, namely AGPase, starch synthase (SS), starch branching enzyme (SBE), and starch debranching enzyme (DBE), are involved in amalgamating the sugar subunits into starch (Hannah and James 2008; Tian et al. 2009). Of these enzymes, AGPase produces ADP glucose and pyrophosphate from glucose-1-phosphate and ATP, which is considered to be the first rate-limiting reaction in starch synthesis (Espada 1962; Smidansky et al. 2002). Enhanced expression of the AGPase gene *ApL3* can improve starch accumulation in Arabidopsis (Wingler et al. 2000). In addition, an *APS* gene encoding a small subunit of AGPase, and the genes *ApL1*, *ApL2*, and *ApL3* encoding large subunits of AGPase in Arabidopsis have been isolated (Villand et al. 1993). Transcript levels of these four genes are differentially regulated by the stimuli such as sugar and light (Sokolov et al. 1998). It has been documented that AGPase genes is responsive to sugars and ABA in last decades (Fritzius 2001; Rook et al. 2001). The possible reason is that ABA affects the synergistic regulation system involved in starch synthesis (Akihiro et al. 2005). However, the regulation of sugars and ABA on expression of AGPase genes is still unknown. Additional experiments prove that large subunit protein is mainly responsible for starch synthesis (Lin et al. 1988; Wang et al. 1997). Two AGPase genes, encoding small subunits of the protein, have been isolated from *Vicia faba*. Although expression of both genes showed high correlation with accumulation of starch, the expression levels of the genes were dissimilar at different stages of development (Weber et al. 1995).

Starch is the major storage compound in the lotus rhizome. The starch content directly affects the quality of processed products. Therefore, starch synthesis is a very important process in lotus. Recently, a lotus Wx gene (*GBSS*), which encodes a granule-bound SS, was isolated and its expression profile was characterized (Lu et al. 2012). In the present study, three genes that encode AGPases were isolated using a homolog cloning method. The enzymatic activity and expression of the three genes were studied. The results of these experiments will contribute to an improved understanding of the processes involved in starch synthesis and aid the production of good-quality lotus root products.

## Methods

### Plant material

Fifteen lotus cultivars that are widely cultivated in China, comprising ‘XSBZ’, ‘Zao Hua’, ‘Su Zhou’, ‘E’lian 6’, ‘Xin1’, ‘JHMRH’, ‘Xin6’, ‘Zao Bai’, ‘XSHZ’, ‘JHDZH’, ‘Zhen Zhu’, ‘Zhong Hua’, ‘L0026’, ‘3735-1’ and ‘Mei Ren Hong’, were grown at the experimental base of aquatic vegetables in Yangzhou University, Jiangsu, China. Plants were provided with 20–25-cm water depth in spring and average temperatures of 30/20 °C (day/night) for the entire growing season (Cheng et al. 2013).

### Measurement of starch content

Starch content of fifteen lotus was measured according to method described by instruction of ‘Guide for modern plant physiology experiments’ edited by Shanghai Institute of Plant Physiology, Chinese Academy of Sciences. Three rhizomes per cultivar were collected and oven dried at 150 °C for 1 h, and then dried to constant weight at 60 °C. The dried material was ground into powder and starch content was determined at 630 nm with a spectrophotometer (UV2800PC, Hengping, Beijng). Amylose content was measured as described by Niu (1990). 0.1 g of the sample was placed into a tube and 10 ml of 0.5 N KOH was added. the amylose content was measured by comparison against a standard curve.

### Estimation of AGPase activity

Activity of AGPase was measured in accordance with the method of Nakamura et al. (1989). A rhizome sample (0.6 g) was rinsed and ground into power in 5 ml of 100 mmol L^−1^ tricine/NaOH solution (pH 8.0) at 0 °C. AGPase activity was measured at 340 nm wavelength.

For statistical analysis, the data were recorded as the mean ± SE of three experiments, with about 10 seedlings per experiment. Statistical analyses were performed using the SPSS software ver. 14.0 (SPSS Inc., Chicago, IL, USA).

### Isolation of *AGP* genes

Total RNA was isolated from ground rhizome samples of the cultivar ‘Mei Ren Hong’ using RNeasy mini kit (QIAGEN, Hilden, Germany). DNaseI was added to eliminate DNA contamination. The quality of RNA was checked with an ultraviolet spectrophotometer (Eppendorf, Hamburg, Germany). Three gene fragments were cloned using a homolog method, and the PCR products were purified by 1 % agarose gel purification kit, following the manufacturer’s instructions (TaKaRa, Tokyo, Japan), and then ligated into the pMD 18-T vector (TaKaRa). Each cloning vector containing the target a gene was transferred into *Escherichia coli* strain DH5α cells. The recombinants were spread onto Luria broth medium containing ampicillin, 5-bromo-4-chloro-3-indolyl-β-d-galactoside, and isopropyl-β-d-thiogalactopyranoside, and incubated at 37 °C overnight. White colonies were chosen, placed in liquid LB medium, and incubated with shaking at 37 °C overnight. After identification of ‘positive’ clones by PCR amplification, plasmid DNA was extracted using a plasmid Purification Kit (TaKaRa). The plasmids were collected and sequenced by Sangon Biotech (Shanghai, China).

Full-length cDNAs of the three cloned genes were obtained with the random amplification of cDNA ends (RACE) method, using the 3′-Full RACE Core Set Ver.2.0 and 5′-Full RACE Core Set (TaKaRa). Gene-specific outer and inner primers were designed in conserved regions of homolog genes from other plants. Nested PCR was performed using different combinations of the gene-specific primers and the RACE primer. The PCR products were purified, then ligated into the pMD18-T cloning vector (TaKaRa), and subsequently sequenced. For 3′-RACE, the first-strand cDNAs were synthesized using M-MLV reverse transcriptase (TaKaRa) with a 3′-RACE adapter primer. Nested PCR, gel purification, and vector ligation were carried out as described above. Primer synthesis and DNA sequencing were performed by Sangon Biotech. Sequence analysis was undertaken using the DNASTAR software. Knowledge of genomic sequence is indeed important to understand the complete features of a gene. Therefore, for cloning of genomic DNA, the leaf (Mei Ren Hong) was used to amplify full-length DNA by PCR method based on information from the obtained cDNA sequences. All primer sequences are listed in Additional file 1: Table S1.

### Gene expression profile analysis

Temporal and spatial expression of the cloned *NnAGP* genes were analyzed using quantitative real-time PCR (qPCR) and semi-quantitative reverse transcription-PCR (semi RT-PCR). For analysis of temporal expression, total RNA of ‘Mei Ren Hong’ was extracted at four stages of rhizome development, namely the stolon period, initial swelling period, middle swelling period, and late swelling period. For spatial expression analysis, total TNA was extracted from five organs (Mei Ren Hong), namely the rhizome, ‘Houba’ leaf (at this stage, rhizome begins to format), leaf petiole of ‘Houba’, terminational leaf (the last leaf of lotus) and terminational petiole.

Seedlings at the four-leaf stage were treated with exogenous sucrose (10 %) and abscisic acid (ABA; 100 μM) for 24 h. The leaf was used to monitor expression of the three cloned genes. After identification of RNA quality, 2 μg of total RNA from each organ was used to synthesize first-strand cDNA using the first cDNA synthesis Kit (Tiangen, China) in accordance with the manufacturer’s instructions (Tiangen, China). The qPCR reaction was performed with a Mx-3000P™ qPCR machine (Stratagene, La Jolla, CA, USA). The SYBR^®^ Green Master Mix was used to determine the mRNA level in accordance with the manufacturer’s instructions (Tiangen, China). The gene-specific primers and internal standard primers (β-actin) are listed in Table 1. The PCR protocol consisted of 35 cycles of 5 min at 94 °C, 30 s at 94 °C, 30 s at 56 °C, 60 s at 72 °C, and final extension for 10 min at 72 °C. The PCR products were confirmed by 1 % agarose gel electrophoresis.

**Table 1:** Identification of starch content (amylose, amylopectin and total starch content) in different varieties of lotus root including XSBZ, Zao Hua, Su Zhou, E′lian 6, Xin1, JHMRH, Xin6, Zao Bai, XSHZ, JHDZH, Zhen Zhu, Zhong Hua, L0026, 3735-1 and Mei Ren Hong

Varieties	Amylose content (%)	Amylopectin content (%)	Total starch content (%)
XSBZ	1.86 ± 0.015j	13.42 ± 0.032d	15.28 ± 0.047gh
Zao Hua	2.41 ± 0.076i	14.51 ± 0.020a	16.92 ± 0.095c
Su Zhou	2.50 ± 0.051i	10.67 ± 0.020k	13.17 ± 0.032j
E′lian 6	2.52 ± 0.006i	12.79 ± 0.021f	15.31 ± 0.017fg
Xin1	2.68 ± 0.015h	13.56 ± 0.060c	16.24 ± 0.045e
JHMRH	2.77 ± 0.072h	13.47 ± 0.032c	16.24 ± 0.040e
Xin6	2.86 ± 0.020g	13.88 ± 0.015b	16.75 ± 0.015d
Zao Bai	2.86 ± 0.115g	14.48 ± 0.023a	17.34 ± 0.096b
XSHZ	3.14 ± 0.015f	12.04 ± 0.021g	15.18 ± 0.010h
JHDZH	3.14 ± 0.015f	13.12 ± 0.057e	16.26 ± 0.044e
Zhen Zhu	3.50 ± 0.025e	9.70 ± 0.095m	13.20 ± 0.120j
Zhong Hua	3.68 ± 0.025d	11.74 ± 0.010h	15.42 ± 0.035f
L0026	3.95 ± 0.044c	10.59 ± 0.006l	14.55 ± 0.042i
3735-1	5.95 ± 0.040b	11.36 ± 0.010j	17.31 ± 0.050b
Mei Rren Hong	6.32 ± 0.021a	11.53 ± 0.046i	18.85 ± 0.067a

## Results

### Starch content in the rhizome of lotus cultivars

Significant differences in amylose, amylopectin and total starch contents of the rhizome were observed among the 15 lotus cultivars. The ranges in amylose, amylopectin and total starch contents observed were 1.86–6.32 %, 9.7–14.51 % and 13.17–18.85 %, respectively. The contents of amylose (11.53 %) and total starch (18.85 %) for ‘Mei Ren Hong’ were significantly higher than those of the other cultivars analyzed, and the lowest total starch content was recorded for ‘Su Zhou’ (13.17 %). The amylose and amylopectin contents (1.86 and 9.70 %) of ‘XSBZ’ and ‘Zhen Zhu’, respectively, were the lowest observed among the cultivars analyzed (Table 1). Therefore, ‘Mei Ren Hong’ was selected to quantify AGPase activity and for gene expression profile analysis.

**Fig. 1:**
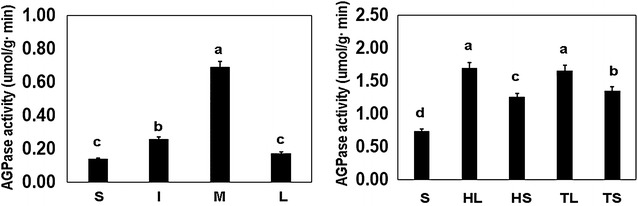
Activity of AGPase at different developmental stages and organs in lotus root. Activity of AGPase enzyme was determined in crude extracts of lotus organs (rhizome, ‘Hou ba’ leaf, ‘Hou ba’ leaf stalk, terminational leaf, terminational leaf stalk) and product organ at different developmental stages (stolon stage, initial swelling stage, middle swelling stage and late swelling stage) as described in “Methods” section

ADP-glucose pyrophosphorylase is the first committed enzyme of the starch biosynthesis pathway, and activity of the enzyme affects starch content in plants. In this study, the cultivar ‘Mei Ren Hong’, which showed the highest starch content in the rhizome, was selected to analyze AGPase activity. Greater activity of AGPase was observed in the ‘houba’ leaf and termination leaf than in the rhizome, ‘houba’ leaf petiole and termination leaf petiole. Activity of AGPase was higher at the middle swelling stage than that the other rhizome developmental stages (Fig. 1).

### Isolation of *AGP* genes

A homolog cloning method combined with the RACE technique was used to isolate full-length cDNAs of three genes from ‘Mei Ren Hong’. Comparison of the gene sequences with sequences lodged in the National Center for Biotechnology Information (NCBI) database through tblaxt biological software confirmed that the cDNAs were full-length sequences of AGPase genes. The three genes showed high similarity with *AGPL1*, *AGPL2* and *AGP*S from other species. Therefore, we designated the genes *NnAGPL1*, *NnAGPL2*, and *NnAGPS*, respectively. The full-length cDNAs of *NnAGPL1*, *NNAGPL2*, and *NnAGPS* were 1587, 1566 and 1945 bp, respectively, consisting of a single open reading frame, and encoded a putative polypeptide of 528, 521 and 524 amino acids (Additional files 2, 3, 4: Figures S1–S3).

**Fig. 2:**
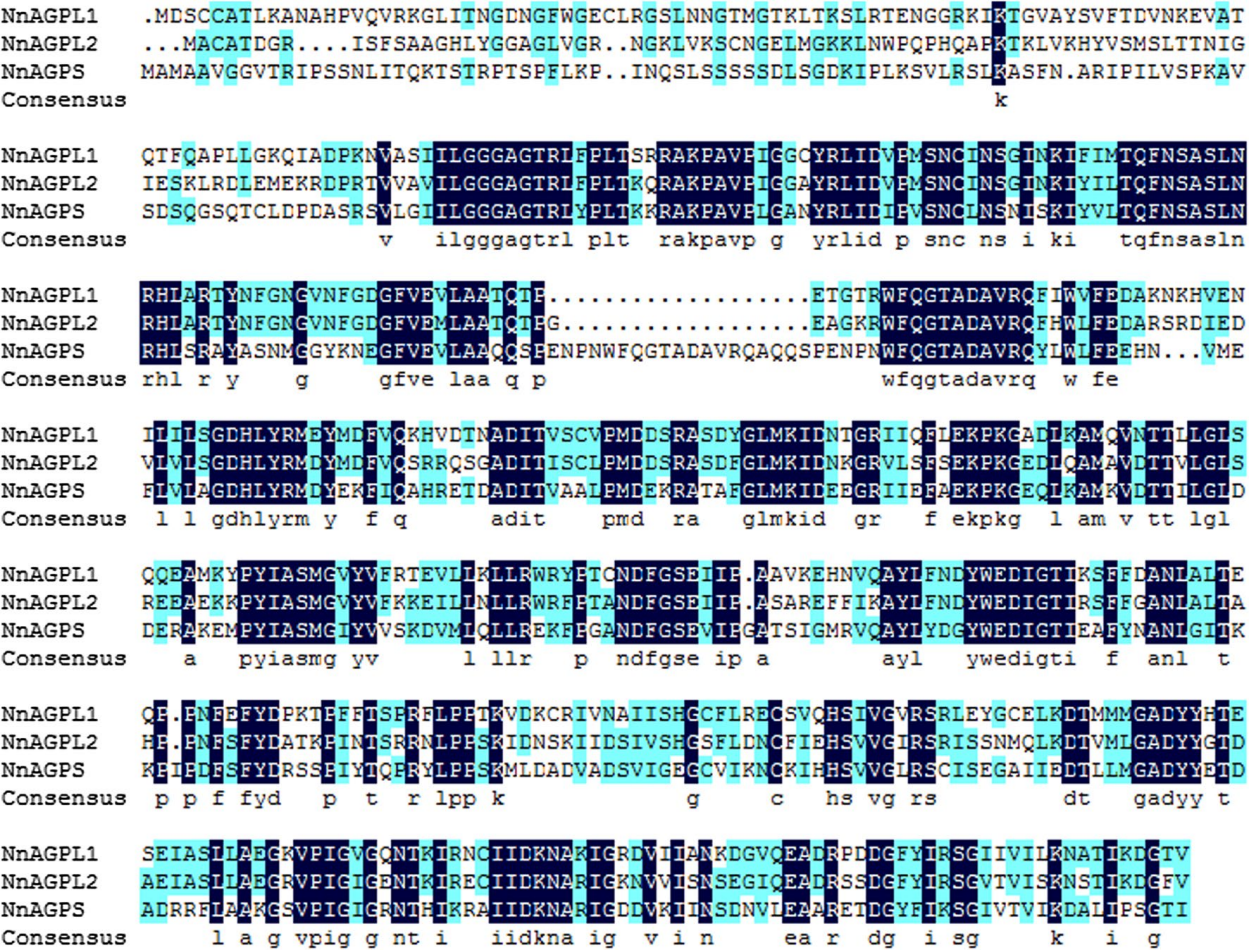
Alignment of the deduced amino acid of NnAGPL1, NnAGPL2 and NnAGPS

**Fig. 3:**
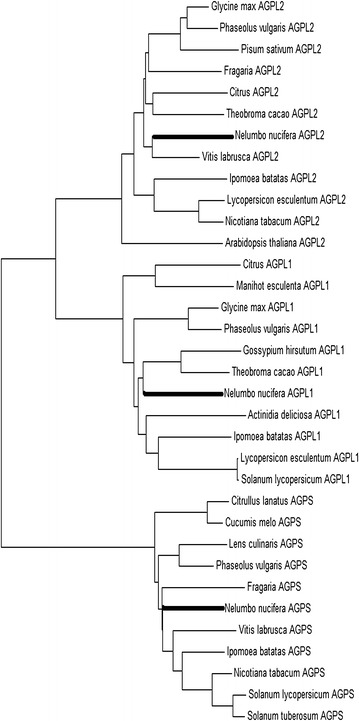
Analysis of phylogenetic tree of plant NnAGP proteins from different species. The unrooted tree was constructed by using MEGA 4.0 software with a neighbor-joining method. The parameters pairwise deletion and JTT (Jones, Taylor, and Thornton) amino acid substitution model were used. Sequences aligned included AGPL1, AGPL 2, AGPL 3, AGPL 4, and AGPS that were identified or predicted from NCBI database

Compared with protein sequences lodged in the NCBI database, *NnAGPL1* showed 79, 79, 81, 79 and 79 % sequence similarity with *AGPL1* from *Prunus persica* (EMJ23720), *Theobroma cacao* (EOY14815), *Populus trichocarpa* (XP_002300758), *Fragaria vesca* (XP_004291856) and *Vitis vinifera* (XP_002281223), respectively. *NnAGPL2* showed 100 % similarity with *AGPL2* of *V. vinifera* (XP_002281069), *Actinidia chinensis* (AFO84093), *Ricinus communis* (XP_002517196), *Theobroma cacao* (EOY05585), and *Glycine max* (XP_003549968), respectively (Fig. 3). *NnAGPS* showed 99, 91, 90, and 90 % similarity with *Nelumbo lutea* (AHZ08828.1), *Oryza sativa* (ACJ86318.1, *Ipomoea batatas* (AFL55401.1), and *Hordeum vulgare* (ABX72229.1), respectively (Additional files 5, 6, 7: Figures S4–S6). In addition, 40.5 % similarity in the deduced amino acid sequences of *NnAGPL1*, *NnAGPL2*, and *NnAGPS* was observed (Fig. 2). In addition, phylogenetic analysis of *NnAGPL1*, *NnAGPL2* and *NnAGPS* with *AGPL1*, *AGPL2* and *AGPS* of other species showed that *NnAGPL1*, *NnAGPL2* and *NnAGPS* seemed to have a distant relationship with *AGPL1*, *AGPL2* and *AGPS*, although they were classified to a group, respectively (Fig. 3).

### Genomic DNA sequence and characterization of *NnAGPL1*, *NNAGPL2*, and *NnAGPS*

Genomic DNA (gDNA) sequences for *NnAGPL1*, *NnAGPL2*, and *NnAGPS* were isolated by PCR method based on information from the obtained cDNA sequences. Information on the primers used is provided in Additional file 1: Table S1. Full length gDNAs of *NnAGPL1*, *NnAGPL2*, and *NnAGPS* were 4841, 11,346 and 4169 bp in length and contained 13, 14 and 8 introns, and 14, 15 and 9 exons, respectively. The seventh, first and first introns in the gDNAs of *NnAGPL1*, *NnAGPL2*, and *NnAGPS*, which were 1049, 1167 and 1175 bp, respectively, were the longest introns, whereas the eighth, first and second introns were the shortest. The first, first, and second exon in *NnAGPL1*, *NnAGPL2*, and *NnAGPS* were the longest exons, whereas the sixth, sixth and seventh exons were the shortest, respectively (Table 2).

**Table 2:** Analysis of gDNA of *NnAGPL1*, *NnAGPL2* and *NnAGPS*

Genes	First exon (bp)	Second exon (bp)	Third exon (bp)	Fourth exon (bp)	Fifth exon (bp)	Sixth exon (bp)	Seventh exon (bp)	Eighth exon (bp)	Ninth exon (bp)	Tenth exon (bp)	Eleventh exon (bp)	Twelfth exon (bp)	Thirteenth exon (bp)	Fourteenth exon (bp)	Fifteenth exon (bp)
*NnAGPL I*	231	129	174	90	84	56	94	186	81	87	105	107	61	102	
*NnAGPL II*	222	114	174	93	84	56	94	113	73	81	87	105	107	61	102
*NnAGPLS*	276	298	270	179	104	112	97	122	125						

Genes	First intron (bp)	Second intron (bp)	Third intron (bp)	Fourth intron (bp)	Fifth intron (bp)	Sixth intron (bp)	Seventh intron (bp)	Eighth intron (bp)	Ninth intron (bp)	Tenth intron (bp)	Eleventh intron (bp)	Twelfth intron (bp)	Thirteenth intron (bp)	Fourteenth intron (bp)
*NnAGPI*	302	256	130	114	93	468	1049	72	149	82	293	116	130	
*NnAGPLII*	1667	348	137	1077	86	615	84	660	198	1645	1136	837	85	1205
*NnAGPL S*	1175	92	144	233	118	155	127	262						

### Expression profiles of *NnAGPL1*, *NnAGPL2*, and *NnAGPS*

**Fig. 4:**
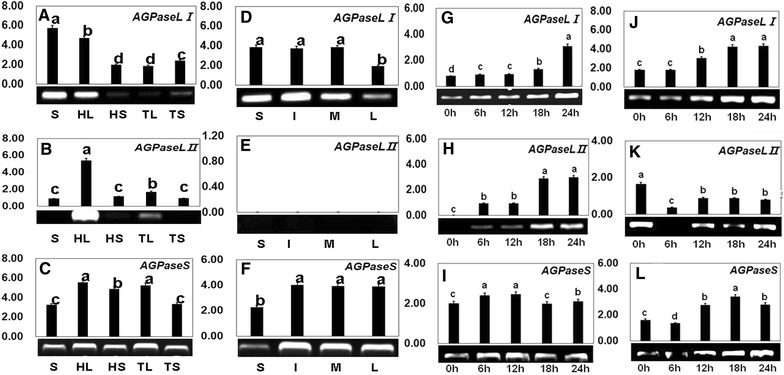
Expression of *NnAGPL1*, *NnAGPL2* and *NnAGPS* with qPCR and semi RT-PCR methods. **A**–**C** Expression analysis of *NnAGPL1*, *NnAGPL2* and *NnAGPS* in different lotus organs; **D**–**F** Expression analysis of *NnAGPL1 NnAGPL2* and *NnAGPS* at different lotus rhizome developmental stages; **G–I** Accumulation of mRNA level of *NnAGPL1*, *NnAGPL2* and *NnAGPS* with treatment of sucrose; **J–L** Expression analysis of *NnAGPL1*, *NnAGPL2* and *NnAGPS* with treatment of ABA

The highest transcript levels for *NnAGPL1* were observed in the rhizome and ‘Houba’ leaf compared with those of the other organs analyzed (Fig. 4A). The mRNA levels detected in the ‘Houba’ leaf, terminational leaf, and terminational leaf stalk were extremely low. Transcripts of *NnAGPL2* were significantly detected in the rhizome (Fig. 4B). The highest levels of *NnAGPS* mRNAs were detected in ‘Houba’ leaf stalk and terminational leaf stalk (Fig. 4C).

The expression profiles of *NnAGPL1*, *NnAGPL2*, and *NnAGPS* in the rhizome showed strong temporal differences. The mRNAs of *NnAGPL1* were detected at all developmental stages, and the transcript level significantly decreased at the late swelling stage (Fig. 4D). No transcripts of *NnAGPL2* were detected in the rhizome at all developmental stages (Fig. 4E). The level of *NnAGPS* transcripts was observed to be enhanced at the initial swelling, middle swelling, and late swelling stages (Fig. 4F).

Lotus seedlings at the four-leaf stage were treated with sucrose and ABA to monitor changes in *NnAGPL1*, *NnAGPL2*, and *NnAGPS* expression. Transcription of the three genes was significantly enhanced after 18 h and 24 h treatment with sucrose (Fig. 4G–I), which indicated that each gene was induced by exogenous sucrose. Transcription of *NnAGPL1* and *NnAGPS* was increased after application of ABA for 12 h, whereas *NnAGPL2* was not affected by ABA treatment and showed constitutive expression (Fig. 4J–L).

## Discussion

### Starch metabolism during rhizome development

Many carbohydrate-rich plants, such as rice, maize, lotus, and potato, are loaded with not only ample nutrients, but are also excellent sources of vitamins A and C and many other vitamins and minerals (Abbott 1999; Guillon and Champ 2002; De Vries and Toenniessen 2002). Among carbohydrates, starch provides not only nutrients, but also energy for metabolism through hydrolysis or oxidation reactions during plant growth (Enes et al. 2006; Gao et al. 1998). In China, lotus root is a popular aquatic vegetable. Starch is the most important component present in the rhizome, constituting on average 10–20 % of the total fresh weight. Therefore, the starch content directly affects the quality of lotus root products, and thus is considered the most important quality-determining substance. The present results showed that starch content differed significantly among lotus cultivars, which directly determined the end use of the rhizome (Table 1). A high starch content in the rhizome is favored in the Yangtze River region for traditional cooking, thus breeding cultivars with a high starch content in the rhizome is important in many regions of China. It is known that AGPase is an important rate-limiting enzyme in the starch biosynthesis pathway, in both photosynthetic and non-photosynthetic tissues (Pettersson and Ryde-Pettersson 1989; Preiss 1991). In the current study, we observed that AGPase activity showed dissimilarities in different organs and at different developmental stages, which reflected the temporal and spatial regulation of starch synthesis in the rhizome (Fig. 1). Therefore, improvement in AGPase activity in the rhizome by bioengineering approaches is likely to be an efficient means of enhancing the starch content.

### Subunits of AGPase

The molecular mass of AGPase in different organisms ranges from 200 to 400 kD (Weber et al. 1995). Bacterial AGPase consists of four subunits, of which the small unit shows a closer relationship than that of large subunit (Preiss 1991; Weber et al. 1995). In plants, AGPase exists as a heterotetramer, consisting of large and small subunits (Preiss 1991). With respect to the subcellular localization, the plant genome contains four types of AGPase subunits, namely a cytosolic small subunit, plastidial small subunit, cytosolic large subunit, and plastidial large subunit (Burton et al. 2002; Iglesias et al. 1994; Smith-White and Preiss 1992). However, Nakamura and Kawaguchi (1992) observed that AGPase contains six different subunits in the developing endosperm of rice. This phenomenon was confirmed by Akihiro et al. (2005), who identified six genes that encode two small subunits and four large subunits in rice. In lotus, AGPase might consist of two large subunits and one small subunit because we isolated only two *NnAGPL*s and one *NnAGPS* from the rhizome on the basis of current genomic information (Additional files 2, 3, 4, 5, 6, 7: Figures S1–S6).

The AGPases localized in the cytosol and plastids are encoded by various genes (Burton et al. 2002; Giroux and Hannah 1994; Johnson et al. 2003). In plants, a mutation in the small subunit of AGPase synthesized in the cytoplasm and targeted for plastids does not affect overall activity of AGPase. This finding suggests that small subunits of AGPase localized in the cytosol and plastids are encoded by different genes (Johnson et al. 2003). It also provides a clue to the low sequence homology observed among AGPases (Burton et al. 2002; Johnson et al. 2003). In the present study the two large subunits of NnAGPase showed about 53 % similarity, and only 40.5 % similarity in the deduced amino acid sequences was observed among *NnAGPL1*, *NnAGPL2*, and *NnAGPS* (Fig. 2). These results suggested that the three genes might be synthesized in different organs or serve different functions during starch synthesis.

### Structure of gDNA of *NnAGPL1*, *NnAGPL2*, and *NnAGPS*

Generally, mRNAs that prematurely terminate translation are regulated by nonsense code to maintain a low abundance. This mechanism is present in all eukaryotes to maintain the mRNA level or alter the encoded proteins (Maquat 1996; Ruiz-Echevarria et al. 1996). The two alleles *Wx* ^*a*^ and *Wx* ^*b*^, which are located at the *waxy* locus, encode starch synthase in cultivated rice. However, their expression profiles are significantly different owing to a different splice site of the first intron, which leads to 10-fold increase in the expression level of *Wx* ^*a*^—compared with that of *Wx* ^*b*^ (Isshiki et al. 1998). The phenomenon of alternative splicing is also common in plants. Different transcripts that encode proteins with functional differences are produced through alternative splicing (Lorkovic et al. 2000). Lotus cultivars are classified into two categories on the basis of the starch content of the rhizome, which determines the end use. In the present study, the cDNAs of *NnAGPL1*, *NnAGPL2*, and *NnAGPS* contained 13, 14 and 8 introns, respectively (Table 2). Interestingly, we determined that the cDNA sequence of *NnAGPL1* and *2*, *NnAGPS* in the two categories of lotus are almost identical. This finding suggests that differences in starch content among cultivars might be determined by alternative splicing or different splice sites.

### Expression of *NnAGP* genes in the plant kingdom

Ample evidence indicates that AGPase, which comprises large and small subunits, plays a critical role in regulating starch synthesis in photosynthetic organs (Preiss and Sivak 1995). *Arabidopsis* mutants for *adg2*, which lacks the large subunit of AGPase, show low AGPase activity. This finding indicates that the large protein subunit is essential for AGPase function in starch synthesis (Lin et al. 1988). Although *adg2* mutants show some enzyme activity, it also suggests that the small subunit might play an important role (Li and Preiss 1992). The above-mentioned reports provide evidence for the importance of both smaller and larger subunits, but that the large subunit makes a greater contribution to AGPase activity (e.g., Johnson et al. 2003; Kang et al. 2010). Kang et al. (2010) observed that two cultivars of winter wheat expressed two identical small subunits, yet the grain starch contents differed. Interestingly, the species with a high starch content in the grain exhibited higher expression levels of the large subunit. These findings suggest that different transcript levels of the large subunit probably contribute to the differences in AGPase activity and rate of starch accumulation (Singh et al. 2001). Over-expression of the large subunit gene can enhance AGPase activity, seed weight, and starch content. Consequently, attention has focused on isolation of the large subunit gene (Li et al. 2010). Analysis of the transcription levels of two large subunit genes was performed with the aim to further explore the mechanisms of starch synthesis in the two types of cultivated lotus that show different starch contents in the rhizome. We observed that *NnAGPL1* showed high transcription levels in the rhizome and ‘Houba’ leaf, whereas transcription of *NnAGPL2* was detected only in the ‘Houba’ leaf (Fig. 4A, B). Expression of *NnAGPL1* and *NnAGPL2* at specific stages of development indicates that they function in distinct metabolic domains in lotus. In addition, the present results confirmed that transcription of *NnAGPL1*, *NnAGPL2*, and *NnAGPS* showed a high degree of consistency during rhizome development, because rhizome formation starts after emergence of the ‘Houba’ leaves. In addition, transcription of *NnAGPL1* and *NnAGPS* was closely associated with rhizome formation because these two genes showed high mRNA levels during rhizome formation (Fig. 4D, F).

RNA gel blot analysis of expression of the large subunit gene of sugar beet indicated that the large subunit and small subunit genes were strongly expressed in sink and source leaves (Müller-Röber et al. 1995). A high level of mRNA accumulation for the large subunit gene was observed in the stem of sweet potato, but it was totally absent in the tuber and photosynthetic leaves (Harn et al. 2000; Lee et al. 1999). These results indicate that some large subunit genes may show specific expression patterns in plants. In the present study, we also observed different levels of expression of *NnAGP1* and *NnAGP2* at different stages of rhizome development (Fig. 4D, E), suggesting that the regulation of AGPase during starch synthesis is complex. Exogenous sugar increases expression of AGPase genes during plant development (Akihiro et al. 2005). Yu et al. (1991) reported that the mRNA level for the α-amylase gene of rice is enhanced under sugar starvation, and decreases under sugar abundance, which indicates that plants regulate starch metabolism via the sucrose level (Akihiro et al. 2005). The expression of *NnAGPL1*, *NnAGPL2*, and *NnAGPS* was induced by exogenous sucrose (Fig. 4G–I), suggesting that sucrose level might directly regulate starch accumulation through changing the expression of *NnAGPL1*, *NnAGLP2*, and *NnAGPS* in lotus. Expression of some AGPase large subunits is regulated by ABA (Rook et al. 2001). Akihiro et al. (2005) considered that higher plants possess an ABA-regulated system to control starch synthesis, because many starch synthesis-related genes are induced by ABA. The expression of *NnAGPL1* was significantly increased after ABA treatment for 18 and 24 h, whereas no change was detected in the transcript levels of *NnAGPL2* and *NnAGPS* (Fig. 4J–L). This result suggests that the role of *NnAGP1* in controlling starch metabolism might be ABA-dependent.

## Conclusions

Based on analysis of starch content and AGPase activity, we isolated three AGPase genes from the lotus rhizome using a homolog cloning method. The genes were designated *NnAGPL1*, *NnAGPL2*, and *NnAGPS*. *NnAGPL1* consists of 14 exons and 13 introns, *NnAGPL2* contains 15 exons and 14 introns, and *NnAGPS* consists of nine exons and eight introns. Analysis of the expression of the genes by qPCR and semi RT-PCR shows that the three genes display significant differences in spatial and temporal expression patterns. We conclude that regulation of AGPase in the context of starch synthesis is extremely complex. The present findings when integrated with bioengineering approaches will assist in improving the quality of lotus root products.

## Additional files

**Additional file 1: Table S1.** Primers for isolation of *NnAGPL1*, *NnAGPL2* and *NnAGPS.*

**Additional file 2: Figure S1.** Cloning of full length cDNA and deduced amino acid of *NnAGPL1.*

**Additional file 3: Figure S2.** Cloning of full length cDNA and deduced amino acid of *NnAGPL2.*

**Additional file 4: Figure S3.** Cloning of full length cDNA and deduced amino acid of *NnAGPS.*

**Additional file 5: Figure S4.** Comparison of *NnAGPL1* against *AGPL1* s of other species.

**Additional file 6: Figure S5.** Comparison of *NnAGPL2* against *AGPL2* s of other species.

**Additional file 7: Figure S6.** Comparison of *NnAGPS* against *AGPS* of other species.
