# Supplementary material for: Activity and expression of ADP-glucose pyrophosphorylase during rhizome formation in lotus (Nelumbo nucifera Gaertn.)
Source: Bot Stud. 2016 Sep 30;57:26. doi: 10.1186/s40529-016-0140-z (PMC5432948; doi:10.1186/s40529-016-0140-z)
Supplement: Supplementary file 1 — Additional file 1: Table S1. Primers for isolation of NnAGPL1, NnAGPL2 and NnAGPS. [file 40529_2016_140_MOESM1_ESM.doc]

| Prime Name  Additional table.1 Primers for cloning of *NnAGPL1,* *NnAGPL2* and *NnAGPS* | Sequence (5'-3') |
| --- | --- |
| 3´RACE (*NnAGPL1*): | Forward primer: 5'-CATTCCAATGAGCAACTG-3'  Forward primer: 5'- GAGCTTTGGAGATGGATTTG-3' |
| 5´RACE (*NnAGPL1*): | Reverse primer: 5'- CGGTTGAGAGAAGCAGAGTTG-3'  Reverse primer: 5'-CGACCTCCTTGTTGACATCTG-3' |
| 3´RACE (*NnAGPL*2): | Forward primer: 5'-TTTGATGGCTACTGGGAGG-3'  Forward primer: 5'- GCAATGCAAGTAGATAC-3' |
| 5´RACE (*NnAGPL*2): | Reverse primer: 5'-TCTCCCTTAGGCTTTTCACTGA-3'  Reverse primer: 5'- AACCACCTTTTACCTGCCTCTC-3' |
| 5´RACE ( *NnAGPS*): | Reverse primer: 5'-TGCGTCCTTCTTCATCAATC-3'  Reverse primer: 5'-CCGAGAAAGGTGGCGATT-3' |
| gDNA primes of *NnAGPL1* | Forward primer: 5'-ATGGATTCTTGTTGTGCGACC-3'  Reverse primer: 5'-CGGTTGAGAGAAGCAGAGTTGA-3' |
|  | Forward primer: 5'-TGATGTCCCAATGAGCAACTG-3'  Reverse primer: 5'-GGCCTGTGTTATCGATCTTCAT-3' |
|  | Forward primer: 5'-CATGTGGACACAAATGCTGATA-3'  Reverse primer: 5'-TTCCAATGTCTTCCCAGTAATC-3' |
|  | Forward primer: 5'-CACAATGTCCAGGCATATCTAT-3'  Reverse primer: 5'-CCTTAAGCTCACACCCGTACT-3' |
|  | Forward primer: 5'-CCACCTACCAAAGTCGACAA-3'  Reverse primer: 5'-GACTCTATATGACGGTACCATC-3' |
| gDNA primes of *NnAGPL*2 | Forward primer: 5'-GAGGAAATGGCGTGCGCCAC-3'  Reverse primer: 5'-GCCTGTTAAGCGATGCAGAGTT-3' |
|  | Forward primer: 5'-GATGTGCCAATGAGCAACTGTA-3'  Reverse primer: 5'-CTGTCATCCATAGGGAGACA-3' |
|  | Forward primer: 5'-GGCAGAGTGGTGCTGATATTAC-3'  Reverse primer: 5'-CTTGCTGAGGCTGGGATTAT-3' |
|  | Forward primer: 5'-GCGTTTTCCTACTGCAAATGA-3'  Reverse primer: 5'-GCTGCTTATTCTTGACCGTAT-3' |
|  | Forward primer: 5'-ACACGGAAGTTTCTTGGATAA-3'  Reverse primer: 5'-ATCATATTACAAATCCATCT-3' TTGAT-3' |
| gDNA primes of *NnAGPS* | Forward primer: 5'-GAAAATCGTCTTCCTCACTC-3'  Reverse primer: 5'-TTCCTGCTCCACCCCCAAGT-3' |
|  | Forward primer: 5'-TACTTGGGGGTGGAGCAGGA-3'  Reverse primer: 5'-CAGTGGCACGCTTTTCATCC-3' |
|  | Forward primer: 5'-TACCGCTGATGCTGTAAGGC-3'  Reverse primer: 5'-CACTTCCGAAATCATTGGCT-3' |
|  | Forward primer: 5'- GGACTTGATGATGAGAGAGC-3'  Reverse primer: 5'-AATCACACACCCCTCACCAA-3' |
|  | Forward primer: ATTGGTGAGGGGTGTGTGAT-3'  Reverse primer: 5'-GTGATTGACCCCCTTACTAC-3' |
|  | Forward primer: 5'-TAAGGGGGTCAATCACTACT-3'  Reverse primer: 5'-GGCAAACAAAATGGTAGATG-3' |
